# The missing hilum: Chronic lobar collapse

**DOI:** 10.1016/j.rmcr.2025.102333

**Published:** 2025-12-01

**Authors:** Thomas Saliba, David Rotzinger, Denis Tack

**Affiliations:** a Centre Hospitalier Universitaire Vaudois (CHUV), Rue du Bugnon 46, 1005, Lausanne, Switzerland; b Hôpital d’Ath (EpiCURA), Rue Maria Thomée 1, 7800, Ath, Belgium

**Keywords:** Chest x-ray, Radiograph, Lobar collapse, Atelectasis, Hilum, Sign

## Abstract

**Background:**

Chronic lobar collapse, or atelectasis, presents a diagnostic challenge due to the subtlety of radiographic findings, particularly when classic signs are absent.

**Case presentation:**

We present the case of a 62-year-old male with emphysema and progressive dyspnoea, whose chest radiographs revealed indirect signs of left lower lobe (LLL) collapse, including a reduced left hilar silhouette, elevated left hemidiaphragm, right lung herniation, and mediastinal shift. These findings were confirmed by CT imaging, which demonstrated retraction of the LLL along the spine, which is an appearance not easily detectable on standard radiographs.

**Discussion:**

Chronic LLL collapse may be obscured due to reduced lobe volume and compensatory changes in surrounding structures. However, displacement and the apparent absence of the left hilum, in the absence of consolidation, can be a secondary sign of chronic LLL collapse. As such it should be taken in conjunction with other secondary signs of lobar collapse to suggest a possible diagnosis and prompt further confirmatory imaging.

**Conclusion:**

Awareness of imaging patterns of lobar collapse, such as the apparent absence of a hilum, is essential for accurate interpretation, particularly in patients without prior lobectomy and when direct signs of consolidation are lacking.

## 1 Introduction

The diagnosis of lobar collapse, also known as atelectasis, is a key responsibility of the radiologist, making the ability to correctly interpret direct and indirect signs essential [1]. Risk factors for atelectasis include general anaesthesia, prolonged bed rest, shallow breathing (for example due to costal pain), and underlying lung disease. There are various possible aetiologies of lobar collapse, including endobronchial obstruction, increased surface tension within the alveoli, loss of negative pressure within the pleural space, or mechanical compression of the lung parenchyma or bronchus [1].

Atelectasis can take many forms, including round atelectasis which is due to pleural disease or linear atelectasis, resulting in band-like collapse [1].

## 2 Case history

**Fig. 1:**
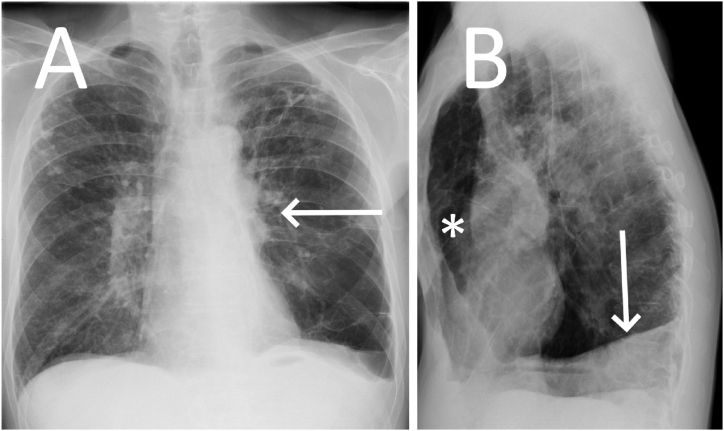
Posteroanterior (A) and lateral (B) chest X-rays demonstrating a small left hilum (A, arrow) when compared to the right side. The lateral chest X-ray shows a loss of lung volume with the ascension of the left hemidiaphragm (B, arrow) and right lung herniation into the anterior mediastinum (B, star).

**Fig. 2:**
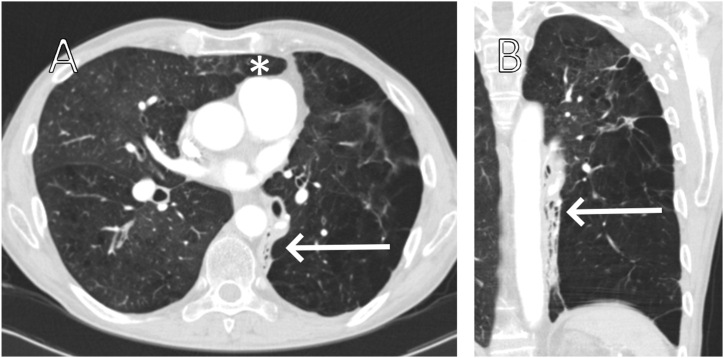
Contrast-enhanced chest CT-scan with pulmonary windowing with axial (A) and coronal (B) reconstructions. This demosntrates left lower lobe (LLL) collapse, with the lobe being retracted along the spine (A, B, arrow). Right lung herniation can be seen (A, star), with the posterior limit of the lung lying against the anterior aspect of the pulmonary trunk.

A 62-year-old man with emphysema and worsening dyspnoea underwent a posteroanterior (PA) chest radiograph showing a smaller left hilum (arrow) compared to the contralateral side (Fig. 1 A). A lateral chest radiograph demonstrated left lung volume loss with hemidiaphragm ascension (arrow), and right lung herniation into the anterior mediastinum (star) delineating the anterior aspect of the pulmonary trunk, indicating mediastinal rotation (Fig. 2 B).

A chest CT-scan with axial (Fig. 2 A) and coronal (Fig. 2 B) reconstructions showed left lower lobe (LLL) collapse, with the lobe being retracted along the spine (arrow), making it occult on x-rays. Complete LLL collapse can be confirmed as the oblique fissure is retracted along the spine (arrow), with the only other lines being present representing the walls of the bullae. If the collapse were only segmental, the fissure would be observed alongside still-aerated parts of the LLL. Right to left mediastinum rotation was also observed. Right lung herniation was present (star), with its posterior limit defined by the anterior aspect of the pulmonary trunk.

The collapsed lobe formed a linear band with an air bronchogram along the left side of the spine, deforming the left hilum and making it less conspicuous on the PA x-ray.

Given the chronic nature of the lower left lobe (LLL) collapse, no treatment was required.

## 3 Discussion

Lung atelectasis is a relatively common reason for obtaining a chest x-ray. When lung atelectasis occurs, a single lobe is involved in the majority of cases, with the left lower lobe making up nearly half of all cases [2].

Chronic lobar collapse can be challenging to diagnose based on x-ray if there is no opacity, leaving the radiologist to rely on secondary signs to infer the diagnosis.

Classically, in a lower lobe collapse, the major fissure is shifted posteriorly towards the costophrenic angle, making it sometimes visible on a lateral radiograph [3].

Differentiating acute from chronic lobar collapse on a radiograph can be challenging. Signs such as a homogenously increased density due to higher soft-tissue density per unit of air, with compensatory hyperinflation of the contralateral side resulting in lower density can help determine acute lobar collapse [2]. These signs can be accompanied by a mediastinal shift towards the collapsed lung and an opacity overlaying the diaphragm [2]. Acute or subacute LLL atelectasis can present as a consolidation located along the spine, with no silhouette sign on the right or left heart border on the PA chest radiograph [4]. The outer limit of this consolidation is an oblique air-fluid linear limit corresponding to the major fissure. The visibility of this fissure on the PA chest view indicates atelectasis of the LLL. In the present case, the major fissure cannot be seen on the PA chest view because it is located a few millimetres from the spine, but the indirect signs of volume loss indicate a high likelihood of chronic LLL collapse.

In chronic collapse, it can sometimes be impossible to differentiate the increased density of the lung from that found in acute collapse, though in some cases the affected area may become so small that it becomes difficult to detect (Robbins). Chronic LLL collapse typically lies posterior and medial to the heart, where it may be partially obscured (Robbins). The mediastinal shift is generally less obvious than in acute cases, with the contralateral lung herniating around the mediastinum and only visible on the lateral chest x-ray [2]. In some cases, the herniation can be so extensive that the collapsed lung appears well aerated; however, an apparent lack of vascular shadows on the side of the herniated lung may provide a clue (Robbins).

A classic but under-recognized sign is the downward shift and ipsilateral hilum shrinking [4]. This sign is less prominent in upper lobe collapse, becausethe lower lobe has the largest bronchi and arteries, which are compressed in cases of collapse [4].

Additional indicators include increased bronchial verticality from posteromedial traction, costophrenic angle blunting due to posterior fissure displacement, and cardiac margin or descending aorta obliteration [4]. Hemidiaphragm elevation or obliteration may also occur as compensation for volume loss [4]. Lateral x-rays can provide further clues, showing increased density over the lower spine [4].

As hilar opacity is mainly due to the pulmonary artery, its displacement due to chronic atelectasis - resulting in the appearance of a missing hilum - is an important indirect sign.

## 4 Conclusion

In a patient without history of prior lobectomy, absence of both the left hilum and consolidation can indicate the presence of chronic lower lobe atelectasis.

## CRediT authorship contribution statement

**Thomas Saliba:** Writing – review & editing, Writing – original draft, Visualization, Validation, Software, Project administration, Funding acquisition, Formal analysis, Conceptualization. **David Rotzinger:** Writing – review & editing, Writing – original draft, Validation, Supervision, Formal analysis, Conceptualization. **Denis Tack:** Writing – review & editing, Writing – original draft, Visualization, Validation, Supervision, Resources, Project administration, Investigation, Formal analysis, Conceptualization.

## Declaration of competing interest

The authors declare that they have no known competing financial interests or personal relationships that could have appeared to influence the work reported in this paper.
